# Oncogenic Mutations and Tumor Microenvironment Alterations of Older Patients With Diffuse Large B-Cell Lymphoma

**DOI:** 10.3389/fimmu.2022.842439

**Published:** 2022-03-25

**Authors:** Yue Zhu, Di Fu, Qing Shi, Ziyang Shi, Lei Dong, Hongmei Yi, Zhenhua Liu, Yan Feng, Qian Liu, Hai Fang, Shu Cheng, Li Wang, Qiang Tian, Pengpeng Xu, Weili Zhao

**Affiliations:** ^1^ Shanghai Institute of Hematology, State Key Laboratory of Medical Genomics, National Research Center for Translational Medicine at Shanghai, Ruijin Hospital, Shanghai Jiao Tong University School of Medicine, Shanghai, China; ^2^ Department of Pathology, Ruijin Hospital, Shanghai Jiao Tong University School of Medicine, Shanghai, China; ^3^ Department of Ultrasound, Ruijin Hospital, Shanghai Jiao Tong University School of Medicine, Shanghai, China; ^4^ State Key Laboratory of Microbial Metabolism, School of Life Sciences and Biotechnology, Shanghai Jiao Tong University, Shanghai, China; ^5^ Laboratory of Molecular Pathology, Pôle de Recherches Sino-Français en Science du Vivant et Génomique, Shanghai, China

**Keywords:** diffuse large B-cell lymphoma, aging, oncogenic mutations, tumor microenvironment, B-cell receptor, histone acetylation

## Abstract

The incidence of diffuse large B-cell lymphoma (DLBCL) increases by age and older DLBCL are commonly related to poor prognosis. However, the clinical and biological features of older DLBCL patients remain to be determined. A total of 2,445 patients with newly diagnosed DLBCL were enrolled for clinical data analysis according to age at diagnosis, with tumor samples of 1,150 patients assessed by DNA sequencing and 385 patients by RNA sequencing. Older DLBCL presented advanced disease stage, elevated serum lactate dehydrogenase, poor performance status, multiple extranodal involvement, high percentage of double expressor subtype, and adverse clinical outcome. According to molecular features, age was positively correlated with the oncogenic mutations of *PIM1*, *MYD88*, *BTG2*, *CD79B*, *TET2*, *BTG1*, *CREBBP*, *TBL1XR1*, and with the MYD88-like genetic subtype. These oncogenic mutations were involved in B-cell receptor/NF-κB signaling, B-cell differentiation, and histone acetylation based on biological functions. Older DLBCL also manifested reduction in CD4^+^ naïve T and CD8^+^ naïve T cells, and also increased recruitment of exhausted T cells and macrophages, leading to immunosuppressive tumor microenvironment. Our work thus contributes to the understanding of aging-related oncogenic mutations and tumor microenvironment alterations in lymphoma progression, and may provide new insights to mechanism-based targeted therapy in DLBCL.

## Introduction

Diffuse large B-cell lymphoma (DLBCL) is the most common aggressive non-Hodgkin lymphoma, with the incidence increased by age. Age >60 at diagnosis is an important risk factor of the International Prognostic Index (IPI), indicating unfavorable clinical outcomes of patients treated by rituximab in combination with cyclophosphamide, doxorubicin, vincristine, and prednisone (R-CHOP) (1). More recently, the National Comprehensive Cancer Network database (NCCN)-IPI has further categorized DLBCL patients into 4 age groups (≤40 years, 41–60 years, 61–75 years, and >75 years) with increasing hazard ratio for inferior overall survival (2). As older DLBCL often present poor baseline health status and intolerance to immunochemotherapy, personalized therapy for older patients remain unmet clinical needs, which may rely on specific molecular features associated with age, especially oncogenic mutations and tumor microenvironment.

As reported, somatic mutations accumulate with age and may be related to tumor progression (3). In DLBCL, *MYD88*, *PIM1*, and *CD79B* mutations were more frequently observed in older patients (4, 5). Although less often occurred in lymphoid malignancies, *TET2* mutations were reported as age-related in other hematological malignancies (6, 7). In addition, immunosenescence contributes to reduced functioning of the immune system with aging (8). As an important feature of immunosenescence, the output of naïve T cells decreases after thymic involution, thereby altering the distribution of CD4^+^ and CD8^+^ naïve T cells in peripheral blood and immune organ (9). Within the tumors, T-cell exhaustion is regulated by immunosuppressive cytokines such as IL-10, and immune cells like macrophages, manifested by loss of effector functions and overexpression of inhibitory receptors like programmed cell death protein-1 (PD-1) (10). Particularly, immunosuppressive M2 macrophages accumulate in older lymphoid tissues and regulate T-cell functions by secreting immunosuppressive cytokines and expressing ligands to inhibitory receptors, and promoting tumor growth and metastasis (11). However, the clinical and biological features of older DLBCL patients remain to be determined.

In this study, we investigated the clinical characteristics and prognostic significance linked to age at diagnosis in a large cohort of 2,445 patients with newly diagnosed DLBCL, and performed genomic and transcriptomic analyses to illustrate the oncogenic mutations and tumor microenvironment alterations associated with older patients.

## Methods

### Patients

**Figure 1:**
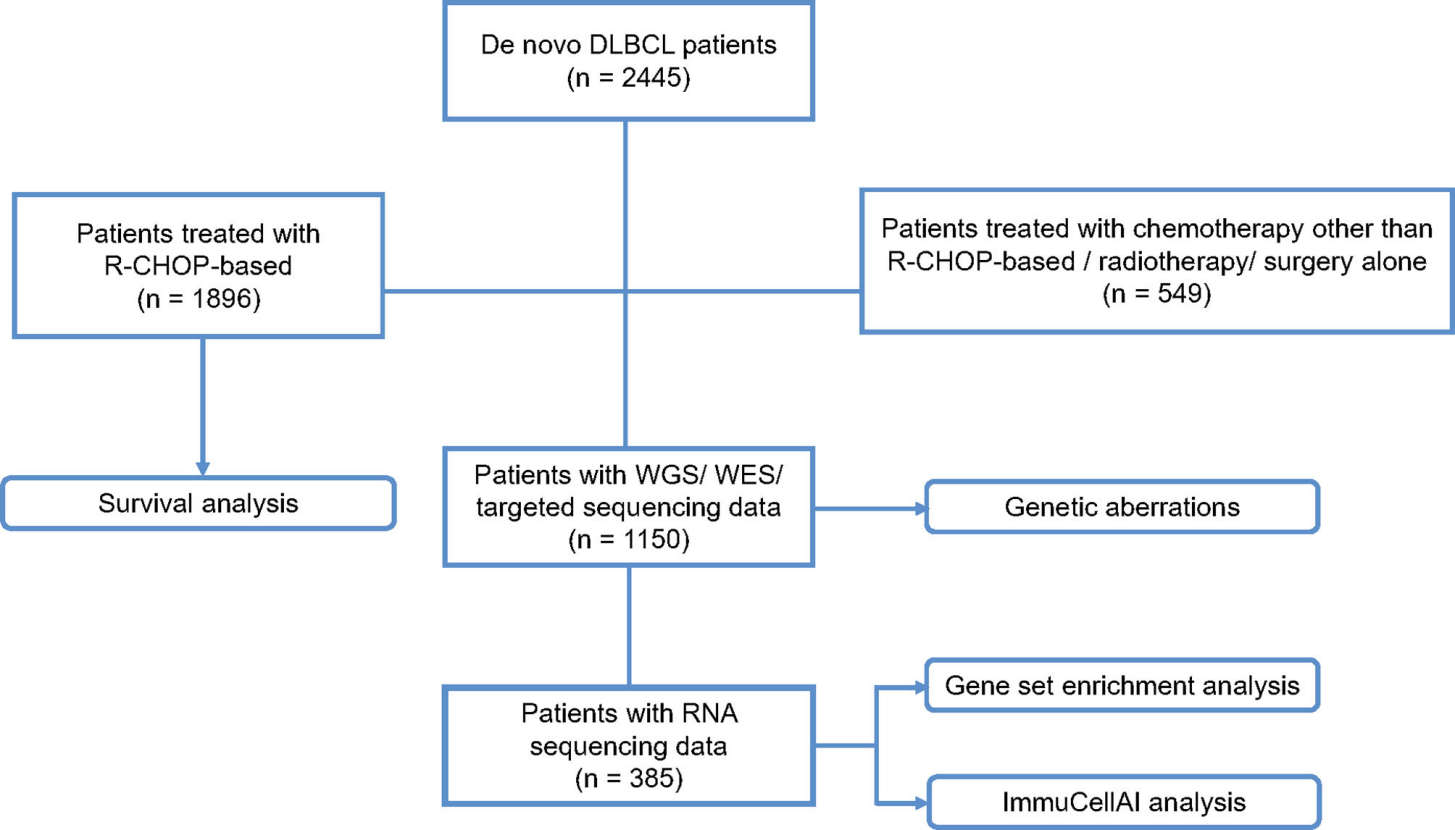
Flow chart of the patient selection and methods. DLBCL, diffuse large B-cell lymphoma; R-CHOP, rituximab, cyclophosphamide, doxorubicin, vincristine and prednisone; WGS, whole genome sequencing; WES, whole exome sequencing.

A flow chart is outlined in **Figure 1** to summarize the patient selection. From September 2002 to April 2021, a total of 2,445 patients with newly diagnosed DLBCL were included, with the last follow-up through August 31, 2021. Histological diagnosis was established based on the World Health Organization (WHO) classification reviewed by two experienced pathologists, LD and HY (12). Survival analysis was performed on 1,896 patients receiving R-CHOP-based immunochemotherapy. DNA and RNA sequencing were performed on 1,150 and 385 patients with available tumor and blood samples, respectively, for detection of genetic aberrations, tumor microenvironmental analysis, and gene set enrichment analysis (GSEA). This study was approved by the Shanghai Rui Jin Hospital Review Board with informed consent obtained in accordance with the Declaration of Helsinki.

### DNA Sequencing

DNA sequencing of 340 patients by whole-genome sequencing (WGS, n = 117) and whole-exome sequencing (WES, n = 223) were performed on available frozen or quality controlled FFPE tumor samples, as reported by our previous studies, along with detailed procedures for DNA sequencing (13–15). Targeted sequencing of 55 lymphoma associated genes were performed among 810 patients with available FFPE tumor samples based on the criteria as previously described (13–15). The mean depth of samples sequenced by WES and WGS was 120.25× (range 50–200×), with an average 97.65% (range 82.64–99.06%) of the target sequence being covered sufficiently deep for variant calling (≥10× coverage). The mean depth of samples sequenced by targeted sequencing was 1,351× (range 505–3,224×), with an average 88.57% (range 59.56–98.26%) of the target sequence being covered sufficiently deep for variant calling (200× coverage). Single nucleotide variations (SNVs) and indels were called by Genome Analysis Toolkit (GATK, v3.4) Haplotype Caller, and GATK Unified Genotyper and mapped to the genome location using the UCSC Genome Browser (http://genome.ucsc.edu) for annotation. The filtration of SNVs and indels was carried out by homemade pipeline with the software mentioned above. Variant allele frequency of mutations included should be over 5%. Mutations were filtered according to the rules listed below. Mutations were preserved if they met the following conditions: 1) sites reported as somatic mutations in our previous studies (13–15); 2) sites verified as somatic mutations by sequencing on paired blood samples; 3) sites commonly considered as hotspot mutations like *MYD88L265P*; 4) sites categorized into tier I and II variants according to the Guideline for Evidence-Based Categorization of Somatic Variants (16); 5) sites not included in the SNP database, or related to hematological malignancies (N >5) as reported in the COSMIC (the Catalogue of Somatic Mutations in Cancer). Mutations were excluded if they met the following conditions: 1) sites verified as germline mutations by sequencing on paired blood samples; 2) sites categorized into tier IV variants according to the Guideline for Evidence-Based Categorization of Somatic Variants; and 3) sites with extraordinarily high frequencies but never reported in previous studies. The mutation data among the 55 genes are listed in **Supplementary Table 1**. Clinical and pathological features of patients with WGS/WES/targeted sequencing data are shown in **Supplementary Table 2**. No statistically significant difference was found in the frequencies of mutations detected by WGS/WES/targeted sequencing called in the components of the cohort after balancing the baseline of clinical and pathological characteristics using propensity score matching, except *DDX3X* (**Supplementary Tables 3** **,** **4**).

Based on the Gene Ontology database, 55 mutated genes were assigned to biological processes, namely, chromatin organization, immune response, cell-cycle/p53, and also oncogenic signaling pathways B-cell receptor (BCR)/NF-κB, JAK-STAT, PI3K-AKT, and Wnt. The list of genes involved in biological function is shown in **Supplementary Table 5**.

### RNA Sequencing, ImmuCellAI Analysis and GSEA Analysis

RNA sequencing was performed on 385 patients with available frozen tumor samples. A total of 361 patients of them were from our previous studies (13–15) and 24 patients were newly analyzed, namely, 98 patients with WGS, 98 with WES, and 189 with targeted sequencing data, respectively. Clinical and pathological features of patients with RNA sequencing data are shown in **Supplementary Tables 6** **–** **8**. RNA was extracted by Trizol and an RNeasy Mini Kit (Qiagen) from available qualified frozen tumor samples of 385 patients. RNA was purified by Ribo-Zero rRNA Removal Kits (Illumina). RNA concentration was assessed on NanoDrop and integrity by an Agilent 2100 Bioanalyzer. RNA libraries were generated with a TruSeq RNA Library Preparation Kit (Illumina) based on instructions of the manufacturer. The concentration and the quality of RNA libraries were controlled by Qubit and BioAnalyzer 2100 system. Paired-end sequencing was performed on Illumina HiSeq sequencer following Illumina-provided protocols. The read pairs were aligned to Refseq hg19 through Burrows‐Wheeler Aligner version 0.7.13‐r1126. The transcript counts table files were generated by the HTSeq (17). Potential false positive results were excluded by visual inspection. R package “sva” was applied to remove batch effect by r 4.0.3. The expressional data related are shown in **Supplementary Table 9**. Cell of origin classification based on gene expression was performed using Lymph2Cx assay (18). Based on raw counts data of RNA sequencing, the infiltration of immune cells including 24 immune cell types were estimated by ImmuCellAI algorithm, a website tool (http://bioinfo.life.hust.edu.cn/web/ImmuCellAI/) (19). Pathway enrichment analysis was performed using GSEA v4.0.1 software as recommended by the GSEA team (http://www.broadinstitute.org/gsea). Pathways were considered of statistical significance with a *P*-value <0.05 and a false discovery rate <0.25.

### Immunohistochemistry

Immunohistochemistry was performed on paraffin sections using antibodies against CD10, BCL6, MUM1, BCL2, and MYC by indirect immunoperoxidase method. Germinal center B-cell-like (GCB) and non-GCB phenotypes were determined with 30% cutoff values of CD10, BCL6, and MUM1, according to Han’s algorithm (20). BCL2/MYC double-expressors were defined by BCL-2 and MYC with cutoff values of 50 and 40%, respectively (12).

### Fluorescence *In-Situ* Hybridization

Fluorescence *in-situ* hybridization of BCL2, BCL6, and MYC rearrangements was performed on paraffin sections with 10% cutoff values. The results in detail are listed in **Supplementary Table 10**.

### Molecular Classification

DLBCL genotypes were identified as described by Lacy et al. using the 47 genes available among 1,150 patients with DNA sequencing data (R code version, https://github.com/ecsg-uoy/DLBCLGenomicSubtyping) (21).

### Statistical Analysis

Pearson’s *χ* ^2^ test or Fisher’s exact test was used to analyze baseline characteristics of patients. Progression-free survival (PFS) was calculated from the date of diagnosis to the date when disease progression or relapse was recognized or the date of last follow-up. Overall survival (OS) was measured from the date of diagnosis to the date of death or the date of last follow-up. Survival functions were analyzed using the Kaplan–Meier method and compared by the log-rank test. Univariate hazard was analyzed using the Cox regression method and the significant variables were then kept in multivariate set. Normalized gene expression in two groups was analyzed using Mann–Whitney *U* test. All statistical analysis was performed by Statistical Package for the Social Sciences (SPSS) 26.0 software. All tests were two-sided, with statistical significance defined as *P <*0.05.

## Results

### Clinical and Pathological Characteristics of DLBCL Patients Based on Age at Diagnosis

Among 2,445 patients with newly diagnosed DLBCL, 1,140 patients were >60 years old and 1,305 patients were ≤60 years old at diagnosis. The clinical characteristics of the patients are listed in **Table 1**. Patients diagnosed at age >60 years were associated with advanced Ann Arbor stage (*P <*0.001), elevated serum lactate dehydrogenase (LDH) (*P <*0.001), poor performance status (*P <*0.001), multiple extranodal involvement (*P* = 0.001), high percentage of double expressor subtype (*P* = 0.012), as compared to those at age ≤60 years. No significant difference was observed between the two age groups, according to gender, cell of origin (Hans), cell of origin (Lymph2Cx), or double/triple-hit.

**Table 1:** Clinical and pathological characteristics of DLBCL patients (n = 2,445).

Characteristics	Age			*P*-value
	≤60 y (n = 1,305)		>60 y (n = 1,140)	
Gender				0.500
Male	708 (54.25%)		634 (55.61%)	
Female	597 (45.75%)		506 (44.39%)	
Ann Arbor stage				<0.001
I–II	694 (53.18%)		516 (45.26%)	
III–IV	611 (46.82%)		624 (54.74%)	
LDH				<0.001
Normal	722 (55.33%)		533 (46.75%)	
Elevated	583 (44.67%)		607 (53.25%)	
ECOG score				<0.001
0–1	1,179 (90.34%)		949 (83.25%)	
≥2	126 (9.66%)		191 (16.75%)	
Extranodal involvement				0.001
0–1	993 (76.09%)		797 (69.91%)	
≥2	312 (23.91%)		343 (30.09%)	
Cell of origin (Hans)				0.199
GCB	395/999 (39.54%)		342/932 (36.70%)	
Non-GCB	604/999 (60.46%)		590/932 (63.30%)	
cell-of-origin (Lymph2Cx)				
ABC	94/202 (46.53%)		98/183 (53.55%)	0.129
GCB	65/202 (32.18%)		42/183 (22.95%)	
unclassified	43/202 (21.29%)		43/183 (23.50%)	
Double expressor				0.012
Yes	180/785 (22.93%)		201/702 (28.63%)	
No	605/785 (77.07%)		501/702 (71.37%)	
Double-hit/triple-hit				0.386
Yes	31/529 (5.86%)		25/535 (4.67%)	
No	498/529 (94.14%)		510/535 (95.33%)	

P-value indicated difference between DLBCL ≤60 years (y) and >60 y.

DLBCL, diffuse large B-cell lymphoma; LDH, lactate dehydrogenase; ECOG, Eastern Cooperative Oncology Group; GCB, germinal center B-cell; ABC, activated B-cell.

### Survival Analysis and Prognostic Significance of DLBCL Patients Based on Age at Diagnosis

**Figure 2:**
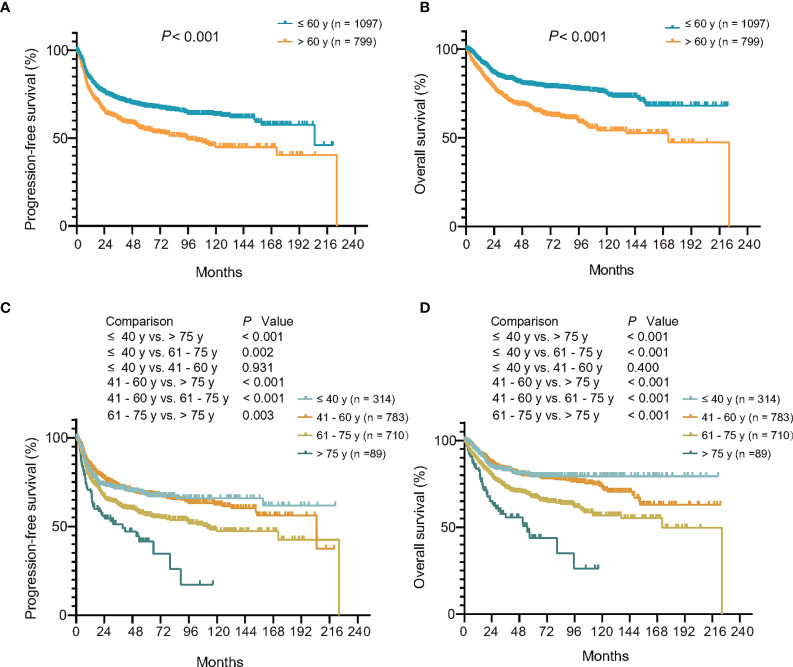
Relationship between age at diagnosis and clinical outcome upon R-CHOP-based immunochemotherapy. **(A, B)** Kaplan–Meier models of progression-free survival **(A)** and overall survival **(B)** according to age at diagnosis of IPI. **(C, D)** Kaplan–Meier models of progression-free survival **(C)** and overall survival **(D)** according to age at diagnosis of NCCN-IPI. R-CHOP, rituximab, cyclophosphamide, doxorubicin, vincristine, and prednisone; IPI, International Prognostic Index; NCCN, National Comprehensive Cancer Network database.

Among 1,896 patients receiving R-CHOP-based immunochemotherapy, the median follow-up time was 55.0 months (0.2–224.2 months). The 3-year PFS and OS rates of patients diagnosed at age >60 years were 60.8 and 71.0%, significantly lower than those of patients at age ≤60 years (72.1%, *P <*0.001; 83.2%, *P <*0.001, respectively) (**Figures 2A, B**). Using univariate analysis, the 5 factors of IPI were of great significance and were included in the multivariate analysis. In the Cox proportional-hazards model, independent prognostic factors of inferior PFS and OS were age (*P* both <0.001), along with Ann Arbor stage (*P* both <0.001), serum LDH (*P* both <0.001), performance status (*P* both <0.001), and multiple extranodal involvement (*P* both <0.05) (**Table 2**). Similar results were observed based on age factor according to NCCN-IPI. The 3-year PFS and OS rates of patients >75 years were 51.2 and 57.6%, significantly lower than those of patients 61–75 years (62.0 and 72.8%), 41–60 years (72.5 and 83.4%), and ≤40 years (71.4 and 82.8%) (**Figures 2C** **, D**).

**Table 2:** Univariate and multivariate analysis for PFS and OS of DLBCL patients (n = 1,896).

	PFS				OS			
	Univariate analysis		Multivariate analysis		Univariate analysis		Multivariate analysis	
	P-value	HR (95% CI)	*P*-value	HR (95% CI)	*P-*value	HR (95% CI)	*P-*value	HR (95% CI)
Age, >60 year	<0.001	1.555 (1.333–1.813)	<0.001	1.394 (1.194–1.628)	<0.001	1.898 (1.577–2.284)	<0.001	1.710 (1.419–2.060)
AA stage, III–IV	<0.001	3.175 (2.693–3.743)	<0.001	2.090 (1.728–2.529)	<0.001	3.506 (2.860–4.297)	<0.001	2.062 (1.634–2.602)
Serum LDH	<0.001	2.994 (2.551–3.513)	<0.001	2.038 (1.714–2.424)	<0.001	3.946 (3.225–4.829)	<0.001	2.652 (2.135–3.294)
ECOG score, ≥2	<0.001	2.772 (2.261–3.399)	<0.001	1.732 (1.404–2.136)	<0.001	3.184 (2.530–4.007)	<0.001	1.887 (1.490–2.390)
ENI, ≥2	<0.001	2.369 (2.015–2.785)	0.018	1.244 (1.039–1.491)	<0.001	2.523 (2.086–3.052)	0.030	1.262 (1.023–1.557)

DLBCL, diffuse large B-cell lymphoma; PFS, progression-free survival; OS, overall survival; HR, hazard ratio; AA, Ann Arbor; LDH, lactate dehydrogenase; ECOG, Eastern Cooperative Oncology Group; ENI, extranodal involvement.

### Oncogenic Mutation Alterations Related to Age at Diagnosis

**Figure 3:**
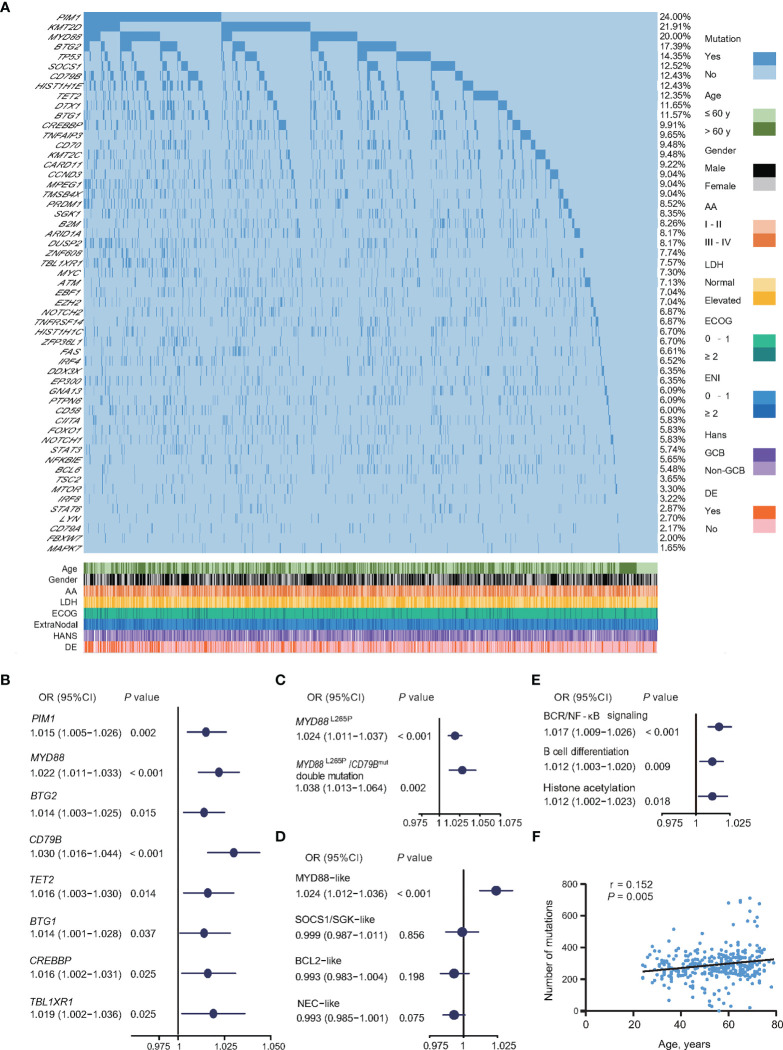
Relationship between oncogenic mutations and age at diagnosis in DLBCL. **(A)** Oncogenic mutations identified by WGS/WES/targeted sequencing in DLBCL patients. **(B)** Univariate logistic regression analysis of oncogenic mutations according to age at diagnosis. **(C)** Univariate logistic regression analysis of *MYD88* ^L265P^ mutations alone or with *CD79B* mutations according to age at diagnosis. **(D)** Univariate logistic regression analysis of genetic subtypes according to age at diagnosis. **(E)** Univariate logistic regression analysis of oncogenic pathways according to age at diagnosis. **(F)** Correlation between the number of oncogenic mutations sequenced by WGS/WES and age at diagnosis in DLBCL. **(B–E)** Odd ratios (OR), 95% confidence intervals (95% CI), and *P*-values are indicated on the left of each forest plot. DLBCL, diffuse large B-cell lymphoma; WGS, whole genome sequencing; WES, whole exome sequencing; AA, Ann Arbor; LDH, lactate dehydrogenase; ECOG, Eastern Cooperative Oncology Group; ENI, extranodal involvement; DE, double expressor; GCB, germinal center B-cell.

Oncogenic mutations closely related to age at diagnosis were analyzed in 1,150 patients, namely, 117 cases by WGS, 223 cases by WES, and 810 cases by targeted sequencing. A total of 55 genes related to the tumorigenesis of DLBCL were analyzed (**Figure 3A**). The association of oncogenic mutations and age at diagnosis among the patients were assessed by univariate logistic regression. Eight genes were significantly correlated with age (**Figure 3B**), namely, *PIM1* (OR = 1.015, 95% CI = 1.005–1.026, *P* = 0.002), *MYD88* (OR = 1.022, 95% CI = 1.011–1.033, *P <*0.001), *BTG2* (OR = 1.014, 95% CI = 1.003–1.025, *P* = 0.015), *CD79B* (OR = 1.030, 95% CI = 1.016–1.044, *P <*0.001), TET2 (OR = 1.016, 95% CI = 1.003-1.030, P = 0.014), *BTG1* (OR = 1.014, 95% CI = 1.001–1.028, *P* = 0.037), *CREBBP* (OR = 1.016, 95% CI = 1.002–1.031, *P* = 0.025), and *TBL1XR1* (OR = 1.019, 95% CI = 1.002–1.036, *P* = 0.025). Meanwhile, the same results were observed on *MYD88* ^L265P^ mutation alone (OR = 1.024, 95% CI = 1.011–1.037, *P <*0.001), or with *CD79B* mutation (OR = 1.038, 95% CI = 1.013–1.064, *P* = 0.002) (**Figure 3C**).

All 1,150 patients were genetically classified (21), namely, MYD88-like (191, 16.6%), SOCS1/SGK1-like (148, 12.9%), BCL2-like (222, 19.3%), and NEC-like subtype (589, 51.2%). MYD88-like subtype was significantly associated with age (OR = 1.024, 95% CI = 1.012–1.036, P <0.001) (**Figure 3D**). Based on biological functions, mutations involving BCR/NF-κB signaling (OR = 1.017, 95% CI = 1.009–1.026, *P <*0.001), B-cell differentiation (OR = 1.012, 95% CI = 1.003–1.020, *P* = 0.009), and histone acetylation (OR = 1.012, 95% CI = 1.002–1.023, *P* = 0.018) were significantly associated with age (**Figure 3E**). The total mutation load in the coding genome also showed positive correlation with age in 340 patients with WGS or WES data (*P* = 0.005, r = 0.152) (**Figure 3F**).

### Tumor Microenvironment Alterations Related to Age at Diagnosis

**Figure 4:**
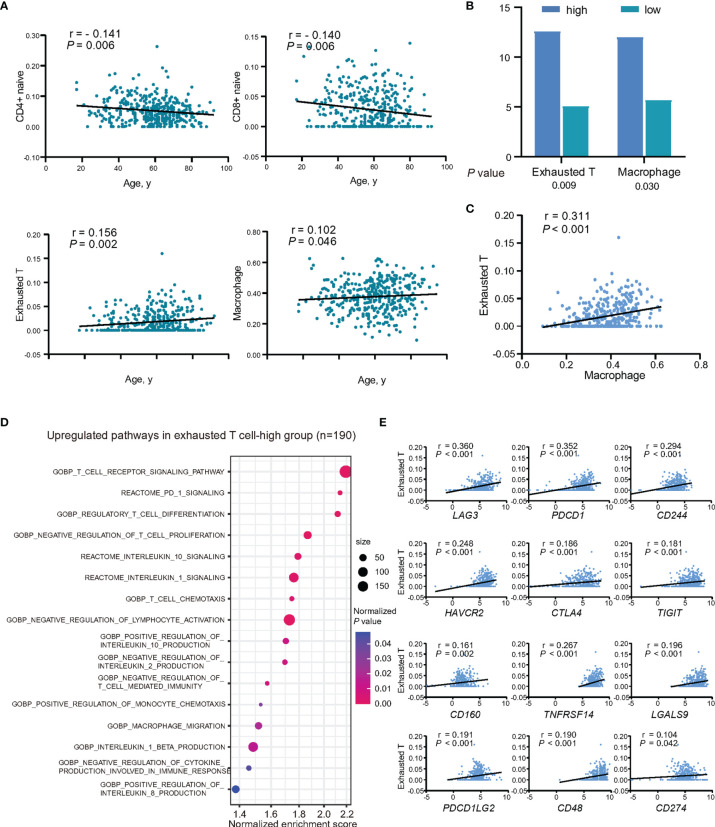
Relationship between intratumor immune cells and age at diagnosis in DLBCL. **(A)** Abundance of immune cell subtypes in patients showing decreased or increased trend with age at diagnosis. **(B)** Mutation rates of *CD79B* between exhausted T-high group and exhausted T-low group, and also between macrophage-high group and macrophage-low group. Lower graph indicates *P*-values. **(C)** Correlation between the abundance of exhausted T cells and macrophages. *P*-value and r-value were indicated in the plot. **(D)** Pathway enrichment analysis in exhausted T-high group (n = 190), as compared to exhausted T-low group (n = 195, *P <*0.05). Color of points indicates normalized *P*-value of upregulated pathways in two groups. Size of points indicates number of genes included in each gene set. **(E)** Correlations between the expression of inhibitory receptors, ligands, and the abundance of exhausted T cells. *P*-values and r-values were indicated in each plot.

The abundance of immune cells in tumor microenvironment was evaluated using RNA sequencing data. The association between the abundance of immune cells and age was estimated using Spearman’s rank correlation. With abundance of CD4^+^ naïve T and CD8^+^ naïve T cells decreased at age (*P* = 0.006 and *P* = 0.006), and abundance of exhausted T cells and macrophages increased with age (*P* = 0.002 and *P* = 0.046) (**Figure 4A**).

As exhausted T cells and macrophages play essential roles in immune evasion, their relations with age-associated mutations and effects on tumor progression were further assessed. All the patients were subsequently divided into two groups according to the median of abundance of the two immune cells, respectively. *CD79B* mutations were found more frequently mutated in the exhausted T-high group and macrophage-high group (*P* = 0.009 and *P* = 0.030) (**Figure 4B**). As revealed by GSEA, BCR signaling pathway was upregulated according to *CD79B* mutations (**Supplementary Figure 1A**). *IL10*, one of the key cytokines associated with T-cell exhaustion and macrophage M2 polarization, was found significantly higher in *CD79B* mutation patients (*P* = 0.001) (**Supplementary Figure 1B**). The abundance of macrophages showed positive linear correlations with the abundance of exhausted T cells (*P <*0.001) (**Figure 4C**).

Correspondingly, GSEA analysis revealed negative regulation of T-cell immunity (T-cell differentiation, lymphocyte activation, and T-cell proliferation, and IL-2 production), upregulation of PD-1 signaling, inhibitory cytokines production and signaling, and also the recruitment of the inhibitory immune cells in exhausted T-high group, as compared to exhausted T-low patients (**Figure 4D**). *IL10*, but not *TGFB1* (TGFβ), showed positive linear correlations with the abundance of exhausted T-cells (*P <*0.001) (**Supplementary Figure 2A**). When analyzing the expression levels of inhibitory receptors and their ligands and the abundance of exhausted T cells, inhibitory receptors, namely, *LAG3*, *PDCD1* (PD-1), *CD244* (2B4), *HAVCR2* (TIM3), *CTLA4*, *TIGIT*, *CD160*, and *BTLA*, and ligands to inhibitory receptors, namely, *TNFRSF14* (HVEM), *LGALS9*, *PDCD1LG2* (PDL2), *CD48*, and *CD274* (PDL1) showed positive linear correlations with the abundance of exhausted T cells (all *P <*0.05) (**Figure 4E** and **Supplementary Figure 2B**). Among them, the expression of *CD244* and *PDCD1* was increased with age (*P* = 0.016 and *P* = 0.031) (**Supplementary Figure 2C**).

**Figure 5:**
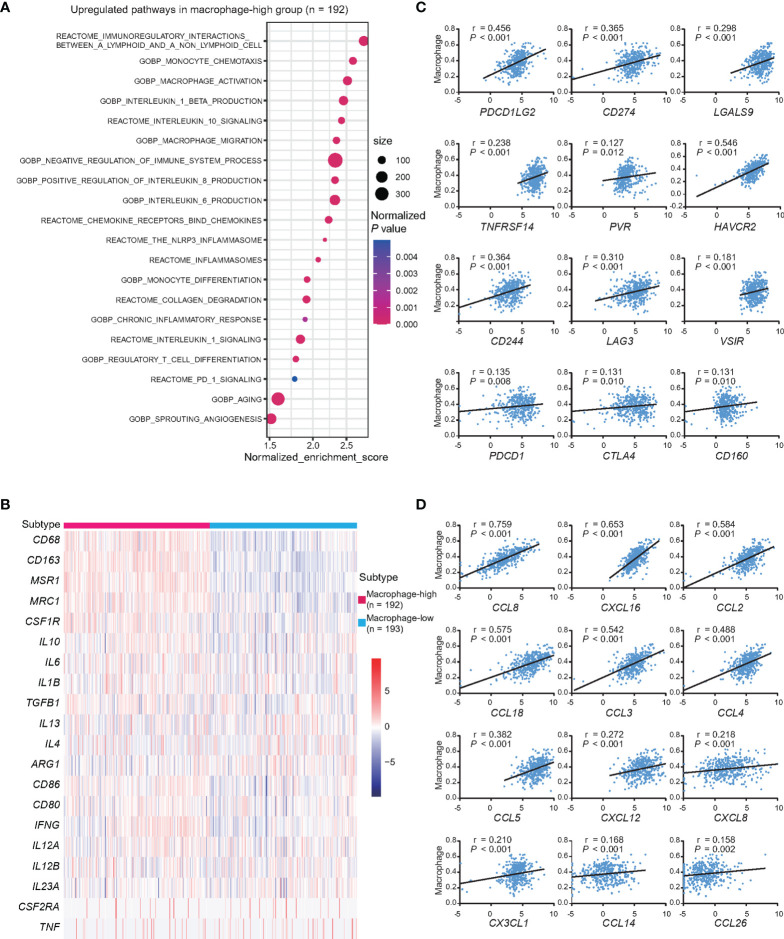
Dysfunctions of macrophages and age at diagnosis in DLBCL. **(A)** Pathway enrichment analysis in macrophage-high group (n = 192), as compared to macrophages-low group (n = 193, *P <*0.05). Color of points indicates normalized *P*-value of upregulated pathways in two groups. Size of points indicates number of genes included in each gene set. **(B)** Heatmap of genes associated with the markers of macrophage M2 and M1 in macrophage-high group (n = 192), as compared to macrophages-low group (n = 193). **(C)** Correlations between the expression of inhibitory receptors, ligands, and the abundance of macrophages. *P*-values and r-values were indicated in each plot. **(D)** Correlations between the expression of chemokines and the abundance of macrophages. *P*-values and r-values were indicated in each plot.

According to the abundance of macrophages, GSEA analysis indicated that upregulation of macrophage recruitment and activation, immunoregulatory interactions of lymphoid and non-lymphoid cells, inflammation, collagen degradation, PD-1 signaling, angiogenesis, aging, and also inhibitory cytokine production and signaling in macrophage-high group, as compared to macrophage-low group (**Figure 5A**). Markers of macrophage M1 and M2 were further compared, showing that M2 markers, namely, *CD163* (log_2_foldchange = 2.693, *P <*0.001), *MSR1* (log_2_foldchange = 2.785, *P <*0.001), and *MRC1* (log_2_foldchange = 1.807, *P <*0.001) were significantly upregulated in macrophage-high group (**Figure 5B**). Meanwhile, cytokines *IL10*, *CSF1*, *IL1B*, *IL6*, and *TGFB1*, which were involved in M2 polarization, showed positive linear correlations with the abundance of macrophages (all *P <*0.001) (**Supplementary Figure 3A**). Since macrophages may contribute to T-cell exhaustion through the expression of ligands to inhibitory receptors, the relationships between the expression levels of inhibitory receptors and their ligands with the abundance of macrophages were analyzed. Among them, ligands to inhibitory receptors, namely, *PDCD1LG2*, *CD274*, *LGALS9*, *TNFRSF14*, *PVR*, and *CD48*, and also inhibitory receptors, namely, *HAVCR2*, *CD244*, *LAG3*, *VSIR* (VISTA), *PDCD1*, *CTLA4*, *CD160*, and *ENTPD1* (CD39), showed positive linear correlations with the abundance of macrophages (all P <0.05) (**Figure 5C** and **Supplementary Figure 3B**). When analyzing the expression levels of chemokines and the abundance of macrophage, chemokines related to macrophage recruitment *CCL8*, *CXCL16*, *CCL2*, *CCL18*, *CCL3*, *CCL4*, *CCL5*, *CXCL12*, *CXCL8*, *CX3CL1*, *CCL14*, and *CCL26* showed positive linear correlations with the abundance of macrophages (all *P <*0.05) (**Figure 5D**). Among these chemokines, the expression of *CCL3* and *CCL5* was increased with age (*P* = 0.024 and *P* = 0.045) (**Supplementary Figure 3C**).

## Discussion

Age at diagnosis is one of the major risk factors in DLBCL, resulting in poor response to R-CHOP-based immunochemotherapy and adverse clinical outcomes, particularly in patients diagnosed over 75 years. In our cohort, older DLBCL presented advanced disease stage, elevated serum LDH, poor performance status, multiple extranodal involvement, high percentage of double expressor subtype, and poor prognosis, which may be resulted from specific biological features.

Based on the genomic data of a large cohort, we observed overall enhanced mutagenesis in the coding region as the age at diagnosis increased in DLBCL, probably due to spontaneous deamination (22). Among oncogenic mutations, eight genes manifested positive association with age at diagnosis. *PIM1*, *MYD88*, and *CD79B* mutations are frequent mutations related to MYD88 genetic subtype and activated B cell-like DLBCL dependent on BCR signaling and constitutive activation of NF-κB pathway, which were found to be enriched in patients that experienced inferior survival and early progression upon R-CHOP treatment (23–26). *BTG2* and *BTG1* were considered as anti-proliferation genes and frequently deleted or mutated in hematological malignancies with aggressive cell behavior and treatment resistance (27). *TET2* mutations impaired enhancer H3K27 acetylation and disrupted transit of B cells through germinal centers, leading to lymphomagenesis (28). *CREBBP* mutations resulted in reduced histone H3 acetylation, enhanced cell proliferation and low expression of MHC II, provoking immune evasion and disease progression (29, 30). *TBL1XR1* mutations co-opted SMRT/HDAC3 repressor complexes toward binding the memory B-cell transcription factor BACH2 and disrupted the differentiation into plasma cells, leading to a striking extranodal immunoblastic lymphoma phenotype (31). Moreover, based on biological functions, mutations involving BCR/NF-κB signaling, B-cell differentiation, and histone acetylation were positively associated with age at diagnosis, which provides potential therapeutic targets, such as BTK inhibitors and epigenetic regulators (32, 33). Together, distinct oncogenic mutations function as important determinators for disease progression in older DLBCL patients and older DLBCL might be considered as a specific entity associated with genetic subtyping.

Tumor microenvironment is also implicated in DLBCL progression. Decreased CD4^+^ naïve T and CD8^+^ naïve T cells were age-related, due to declined production of new T cells from involuted and nonfunctional thymus and results in reduced richness of the TCR repertoire in aging populations (34). Exhausted T cells and macrophages were increased in older patients and related to age-related mutations, particularly *CD79B*. As mechanism of action, *CD79B* mutations contributed to activation of BCR signaling and resulted in upregulated expression of *IL10*, a key immunosuppressive cytokine linked to T-cell exhaustion and macrophage M2 polarization (35, 36). Higher abundance of exhausted T cells presented the suppression of T-cell immunity and upregulation of PD-1 signaling, related to recruitment of immunosuppressive cells and production of immunosuppressive cytokines. The positive correlation between the expression of *IL10* and the abundance of exhausted T cells pointed out that IL10 might be involved in T-cell exhaustion in DLBCL (37). Meanwhile, several inhibitory receptors and ligands were positively correlated to the abundance of exhausted T cells, among which *CD244* and *PDCD1* showed correlation to age (10, 38). Indeed, the inhibitory receptors co-expressed at high levels on T cells and synergistically mediated T-cell exhaustion and dysfunction with the ligands on tumor cells and antigen presenting cells, which implies combinations of agents blocking inhibitory receptors may function in older DLBCL patients to overcome T-cell exhaustion and to restore anti-tumor immunity.

Macrophages constitute a major component of tumor microenvironment and are involved in cancer progression, metastasis and immune evasion, creating an immunosuppressive microenvironment through secretion of anti-inflammatory cytokines, expression of PD-L1, and recruitment of Th2 and Treg cells (39, 40). This was in keeping with the results by pathway analysis that higher abundance of macrophages was associated with the enrichment pathways of negative regulation of immune, chronic inflammatory response, collagen degradation, angiogenesis, and PD-1 signaling in macrophage-high DLBCL. Importantly, higher abundance of macrophages also manifested increased expression of markers of immunosuppressive macrophage M2, namely, *CD163*, *MSR1*, and *MRC1* (41–43). The positive correlation between the expression of cytokines of M2 polarization and the abundance of macrophages suggested that *IL10*, *CSF1*, *IL1B*, *IL6*, and *TGFB1* could involve in M2 polarization in DLBCL (44–48). As macrophages was a major source of immunosuppressive cytokines and showed positive correlation to inhibitory receptors and ligands, the abundance of macrophages was in positive correlation with exhausted T cells and could contribute to age-related immunosuppression in DLBCL (40, 49). Several chemokines related to macrophage chemotaxis and immunosuppressive function increased with macrophage, and among them, *CCL3* and *CCL5* are secreted factors of senescence-associated secretory phenotype and increased with age and age-associated macrophage recruitment (50). Macrophage infiltrations were linked to poor prognosis in DLBCL, suggesting inhibition of macrophages may also be alternative immunomodulatory strategy in DLBCL (14).

However, there was a difference in distribution of clinical features between western and Chinese DLBCL populations according to previous reports and our cohort, namely, age at diagnosis (median age: 62–66 years vs. 53–59 years), Ann Arbor stage (III–IV: 54.1–62.8% vs. 35.2–57.4%), and performance status (Eastern Cooperative Oncology Group score ≥2: 16.7–37.3% vs. 13.0–19.9%) (21, 51–60). Future epidemiology studies are necessary to elucidate the different characteristics between western and eastern countries, which may provide better understanding of targeted therapeutic approaches in DLBCL.

In conclusion, older DLBCL could be a specific entity with unfavorable clinical and molecular features, represented by accumulation of oncogenic mutations and immunosuppressive tumor microenvironment alterations. Thus, clinical studies on immunotherapies warrant further investigation on future mechanism-based treatment in older DLBCL.

## Data Availability Statement

The datasets including WGS, WES and RNA sequencing data were deposited on The National Omics Data Encyclopedia (https://www.biosino.org/node) in project OEP001143 and OEP001040.

## Ethics Statement

The studies involving human participants were reviewed and approved by the Shanghai Rui Jin Hospital Review Board. Written informed consent to participate in this study was provided by the participants.

## Author Contributions

WLZ, PPX, QT, LW, and SC designed and supervised the study. YZ collected and analyzed the data, and wrote the manuscript. DF, QS, and ZYS recruited patients, collected study data, and prepared biological samples. LD and HMY reviewed the histopathologic diagnoses. ZHL acquired the biological samples. YF, QL and HF provided technical support for bioinformatic analysis. All authors listed have made a substantial, direct, and intellectual contribution to the work and approved it for publication.

## Conflict of Interest

The authors declare that the research was conducted in the absence of any commercial or financial relationships that could be construed as a potential conflict of interest.

## Publisher’s Note

All claims expressed in this article are solely those of the authors and do not necessarily represent those of their affiliated organizations, or those of the publisher, the editors and the reviewers. Any product that may be evaluated in this article, or claim that may be made by its manufacturer, is not guaranteed or endorsed by the publisher.

## References

[1] Project INHsLPF. A Predictive Model for Aggressive non-Hodgkin's Lymphoma. N Engl J Med (1993) 329(14):987–94. doi: 10.1056/NEJM199309303291402 8141877

[2] Zhou Z Sehn LH Rademaker AW Gordon LI Lacasce AS Crosby-Thompson A. An Enhanced International Prognostic Index (NCCN-IPI) for Patients With Diffuse Large B-Cell Lymphoma Treated in the Rituximab Era. Blood (2014) 123(6):837–42. doi: 10.1182/blood-2013-09-524108 PMC5527396 24264230

[3] Vijg J Dong X. Pathogenic Mechanisms of Somatic Mutation and Genome Mosaicism in Aging. Cell (2020) 182(1):12–23. doi: 10.1016/j.cell.2020.06.024 32649873 PMC7354350

[4] Di M Huntington SF Olszewski AJ. Challenges and Opportunities in the Management of Diffuse Large B-Cell Lymphoma in Older Patients. Oncologist (2021) 26(2):120–32. doi: 10.1002/onco.13610 PMC7873335 33230948

[5] Kim Y Ju H Kim DH Yoo HY Kim SJ Kim WS. CD79B and MYD88 Mutations in Diffuse Large B-Cell Lymphoma. Hum Pathol (2014) 45(3):556–64. doi: 10.1016/j.humpath.2013.10.023 24444466

[6] Xie M Lu C Wang J McLellan MD Johnson KJ Wendl MC. Age-Related Mutations Associated With Clonal Hematopoietic Expansion and Malignancies. Nat Med (2014) 20(12):1472–8. doi: 10.1038/nm.3733 PMC4313872 25326804

[7] Niroula A Sekar A Murakami MA Trinder M Agrawal M Wong WJ. Distinction of Lymphoid and Myeloid Clonal Hematopoiesis. Nat Med (2021) 27(11):1921–7. doi: 10.1038/s41591-021-01521-4 PMC8621497 34663986

[8] Lian J Yue Y Yu W Zhang Y. Immunosenescence: A Key Player in Cancer Development. J Hematol Oncol (2020) 13(1):151. doi: 10.1186/s13045-020-00986-z 33168037 PMC7653700

[9] Kasakovski D Xu L Li Y. T Cell Senescence and CAR-T Cell Exhaustion in Hematological Malignancies. J Hematol Oncol (2018) 11(1):91. doi: 10.1186/s13045-018-0629-x 29973238 PMC6032767

[10] Wherry EJ Kurachi M. Molecular and Cellular Insights Into T Cell Exhaustion. Nat Rev Immunol (2015) 15(8):486–99. doi: 10.1038/nri3862 PMC4889009 26205583

[11] Jackaman C Nelson DJ. Are Macrophages, Myeloid Derived Suppressor Cells and Neutrophils Mediators of Local Suppression in Healthy and Cancerous Tissues in Aging Hosts? Exp Gerontol (2014) 54:53–7. doi: 10.1016/j.exger.2013.11.009 24291067

[12] Swerdlow SH Campo E Pileri SA Harris NL Stein H Siebert R. The 2016 Revision of the World Health Organization Classification of Lymphoid Neoplasms. Blood (2016) 127(20):2375–90. doi: 10.1182/blood-2016-01-643569 PMC4874220 26980727

[13] Shen R Xu PP Wang N Yi HM Dong L Fu D. Influence of Oncogenic Mutations and Tumor Microenvironment Alterations on Extranodal Invasion in Diffuse Large B-Cell Lymphoma. Clin Transl Med (2020) 10(7):e221. doi: 10.1002/ctm2.221 33252851 PMC7685246

[14] Huang YH Cai K Xu PP Wang L Huang CX Fang Y. CREBBP/EP300 Mutations Promoted Tumor Progression in Diffuse Large B-Cell Lymphoma Through Altering Tumor-Associated Macrophage Polarization *via* FBXW7-NOTCH-CCL2/CSF1 Axis. Signal Transduct Target Ther (2021) 6(1):10. doi: 10.1038/s41392-020-00437-8 33431788 PMC7801454

[15] Qin W Fu D Shi Q Dong L Yi H Huang H. Molecular Heterogeneity in Localized Diffuse Large B-Cell Lymphoma. Front Oncol (2021) 11:638757. doi: 10.3389/fonc.2021.638757 34557402 PMC8454464

[16] Li MM Datto M Duncavage EJ Kulkarni S Lindeman NI Roy S. Standards and Guidelines for the Interpretation and Reporting of Sequence Variants in Cancer: A Joint Consensus Recommendation of the Association for Molecular Pathology, American Society of Clinical Oncology, and College of American Pathologists. J Mol Diagn (2017) 19(1):4–23. doi: 10.1016/j.jmoldx.2016.10.002 27993330 PMC5707196

[17] Anders S Pyl PT Huber W. HTSeq–a Python Framework to Work With High-Throughput Sequencing Data. Bioinformatics (2015) 31(2):166–9. doi: 10.1093/bioinformatics/btu638 PMC4287950 25260700

[18] Scott DW Wright GW Williams PM Lih CJ Walsh W Jaffe ES. Determining Cell-of-Origin Subtypes of Diffuse Large B-Cell Lymphoma Using Gene Expression in Formalin-Fixed Paraffin-Embedded Tissue. Blood (2014) 123(8):1214–7. doi: 10.1182/blood-2013-11-536433 PMC3931191 24398326

[19] Miao YR Zhang Q Lei Q Luo M Xie GY Wang H. ImmuCellAI: A Unique Method for Comprehensive T-Cell Subsets Abundance Prediction and its Application in Cancer Immunotherapy. Adv Sci (Weinh) (2020) 7(7):1902880. doi: 10.1002/advs.201902880 32274301 PMC7141005

[20] Hans CP Weisenburger DD Greiner TC Gascoyne RD Delabie J Ott G. Confirmation of the Molecular Classification of Diffuse Large B-Cell Lymphoma by Immunohistochemistry Using a Tissue Microarray. Blood (2004) 103(1):275–82. doi: 10.1182/blood-2003-05-1545 14504078

[21] Lacy SE Barrans SL Beer PA Painter D Smith AG Roman E. Targeted Sequencing in DLBCL, Molecular Subtypes, and Outcomes: A Haematological Malignancy Research Network Report. Blood (2020) 135(20):1759–71. doi: 10.1182/blood.2019003535 PMC7259825 32187361

[22] Chapuy B Stewart C Dunford AJ Kim J Kamburov A Redd RA. Molecular Subtypes of Diffuse Large B Cell Lymphoma are Associated With Distinct Pathogenic Mechanisms and Outcomes. Nat Med (2018) 24(5):679–90. doi: 10.1038/s41591-018-0016-8 PMC6613387 29713087

[23] Hartert KT Wenzl K Krull JE Manske M Sarangi V Asmann Y. Targeting of Inflammatory Pathways With R2CHOP in High-Risk DLBCL. Leukemia (2021) 35(2):522–33. doi: 10.1038/s41375-020-0766-4 PMC7483252 32139889

[24] Wilson WH Young RM Schmitz R Yang Y Pittaluga S Wright G. Targeting B Cell Receptor Signaling With Ibrutinib in Diffuse Large B Cell Lymphoma. Nat Med (2015) 21(8):922–6. doi: 10.1038/nm.3884 PMC8372245 26193343

[25] Ma J Yan Z Zhang J Zhou W Yao Z Wang H. A Genetic Predictive Model for Precision Treatment of Diffuse Large B-Cell Lymphoma With Early Progression. Biomark Res (2020) 8:33. doi: 10.1186/s40364-020-00214-3 32864130 PMC7448459

[26] Pedrosa L Fernandez-Miranda I Perez-Callejo D Quero C Rodriguez M Martin-Acosta P. Proposal and Validation of a Method to Classify Genetic Subtypes of Diffuse Large B Cell Lymphoma. Sci Rep (2021) 11(1):1886. doi: 10.1038/s41598-020-80376-0 33479306 PMC7820010

[27] Yuniati L Scheijen B van der Meer LT van Leeuwen FN. Tumor Suppressors BTG1 and BTG2: Beyond Growth Control. J Cell Physiol (2019) 234(5):5379–89. doi: 10.1002/jcp.27407 PMC6587536 30350856

[28] Dominguez PM Ghamlouch H Rosikiewicz W Kumar P Beguelin W Fontan L. TET2 Deficiency Causes Germinal Center Hyperplasia, Impairs Plasma Cell Differentiation, and Promotes B-Cell Lymphomagenesis. Cancer Discov (2018) 8(12):1632–53. doi: 10.1158/2159-8290.CD-18-0657 PMC6279514 30274972

[29] Hashwah H Schmid CA Kasser S Bertram K Stelling A Manz MG. Inactivation of CREBBP Expands the Germinal Center B Cell Compartment, Down-Regulates MHCII Expression and Promotes DLBCL Growth. Proc Natl Acad Sci USA (2017) 114(36):9701–6. doi: 10.1073/pnas.1619555114 PMC5594639 28831000

[30] Mondello P Tadros S Teater M Fontan L Chang AY Jain N. Selective Inhibition of HDAC3 Targets Synthetic Vulnerabilities and Activates Immune Surveillance in Lymphoma. Cancer Discov (2020) 10(3):440–59. doi: 10.1158/2159-8290.CD-19-0116 PMC7275250 31915197

[31] Venturutti L Teater M Zhai A Chadburn A Babiker L Kim D. TBL1XR1 Mutations Drive Extranodal Lymphoma by Inducing a Pro-Tumorigenic Memory Fate. Cell (2020) 182(2):297–316.e27. doi: 10.1016/j.cell.2020.05.049 32619424 PMC7384961

[32] Dunleavy K Erdmann T Lenz G. Targeting the B-Cell Receptor Pathway in Diffuse Large B-Cell Lymphoma. Cancer Treat Rev (2018) 65:41–6. doi: 10.1016/j.ctrv.2018.01.002 29549872

[33] Hogg SJ Beavis PA Dawson MA Johnstone RW. Targeting the Epigenetic Regulation of Antitumour Immunity. Nat Rev Drug Discov (2020) 19(11):776–800. doi: 10.1038/s41573-020-0077-5 32929243

[34] Cunha LL Perazzio SF Azzi J Cravedi P Riella LV. Remodeling of the Immune Response With Aging: Immunosenescence and Its Potential Impact on COVID-19 Immune Response. Front Immunol (2020) 11:1748. doi: 10.3389/fimmu.2020.01748 32849623 PMC7427491

[35] Li L Zhang J Chen J Xu-Monette ZY Miao Y Xiao M. B-Cell Receptor-Mediated NFATc1 Activation Induces IL-10/STAT3/PD-L1 Signaling in Diffuse Large B-Cell Lymphoma. Blood (2018) 132(17):1805–17. doi: 10.1182/blood-2018-03-841015 PMC6634963 30209121

[36] Davis RE Ngo VN Lenz G Tolar P Young RM Romesser PB. Chronic Active B-Cell-Receptor Signalling in Diffuse Large B-Cell Lymphoma. Nature (2010) 463(7277):88–92. doi: 10.1038/nature08638 20054396 PMC2845535

[37] Brooks DG Trifilo MJ Edelmann KH Teyton L McGavern DB Oldstone MB. Interleukin-10 Determines Viral Clearance or Persistence In Vivo. Nat Med (2006) 12(11):1301–9. doi: 10.1038/nm1492 PMC2535582 17041596

[38] Blackburn SD Shin H Haining WN Zou T Workman CJ Polley A. Coregulation of CD8+ T Cell Exhaustion by Multiple Inhibitory Receptors During Chronic Viral Infection. Nat Immunol (2009) 10(1):29–37. doi: 10.1038/ni.1679 19043418 PMC2605166

[39] DeNardo DG Ruffell B. Macrophages as Regulators of Tumour Immunity and Immunotherapy. Nat Rev Immunol (2019) 19(6):369–82. doi: 10.1038/s41577-019-0127-6 PMC7339861 30718830

[40] Jackaman C Tomay F Duong L Abdol Razak NB Pixley FJ Metharom P. Aging and Cancer: The Role of Macrophages and Neutrophils. Ageing Res Rev (2017) 36:105–16. doi: 10.1016/j.arr.2017.03.008 28390891

[41] Gundra UM Girgis NM Ruckerl D Jenkins S Ward LN Kurtz ZD. Alternatively Activated Macrophages Derived From Monocytes and Tissue Macrophages are Phenotypically and Functionally Distinct. Blood (2014) 123(20):e110-22. doi: 10.1182/blood-2013-08-520619 24695852 PMC4023427

[42] Mantovani A Sozzani S Locati M Allavena P Sica A. Macrophage Polarization: Tumor-Associated Macrophages as a Paradigm for Polarized M2 Mononuclear Phagocytes. Trends Immunol (2002) 23(11):549–55. doi: 10.1016/S1471-4906(02)02302-5 12401408

[43] Song Q Hawkins GA Wudel L Chou PC Forbes E Pullikuth AK. Dissecting Intratumoral Myeloid Cell Plasticity by Single Cell RNA-Seq. Cancer Med (2019) 8(6):3072–85. doi: 10.1002/cam4.2113 PMC6558497 31033233

[44] Jung M Ma Y Iyer RP DeLeon-Pennell KY Yabluchanskiy A Garrett MR. IL-10 Improves Cardiac Remodeling After Myocardial Infarction by Stimulating M2 Macrophage Polarization and Fibroblast Activation. Basic Res Cardiol (2017) 112(3):33. doi: 10.1007/s00395-017-0622-5 28439731 PMC5575998

[45] Pyonteck SM Akkari L Schuhmacher AJ Bowman RL Sevenich L Quail DF. CSF-1R Inhibition Alters Macrophage Polarization and Blocks Glioma Progression. Nat Med (2013) 19(10):1264–72. doi: 10.1038/nm.3337 PMC3840724 24056773

[46] Das S Shapiro B Vucic EA Vogt S Bar-Sagi D. Tumor Cell-Derived IL1beta Promotes Desmoplasia and Immune Suppression in Pancreatic Cancer. Cancer Res (2020) 80(5):1088–101. doi: 10.1158/0008-5472.CAN-19-2080 PMC7302116 31915130

[47] Weng YS Tseng HY Chen YA Shen PC Al Haq AT Chen LM. MCT-1/miR-34a/IL-6/IL-6R Signaling Axis Promotes EMT Progression, Cancer Stemness and M2 Macrophage Polarization in Triple-Negative Breast Cancer. Mol Cancer (2019) 18(1):42. doi: 10.1186/s12943-019-0988-0 30885232 PMC6421700

[48] Liu F Qiu H Xue M Zhang S Zhang X Xu J. MSC-Secreted TGF-Beta Regulates Lipopolysaccharide-Stimulated Macrophage M2-Like Polarization *via* the Akt/FoxO1 Pathway. Stem Cell Res Ther (2019) 10(1):345. doi: 10.1186/s13287-019-1447-y 31771622 PMC6878630

[49] Farhood B Najafi M Mortezaee K. CD8(+) Cytotoxic T Lymphocytes in Cancer Immunotherapy: A Review. J Cell Physiol (2019) 234(6):8509–21. doi: 10.1002/jcp.27782 30520029

[50] Schafer MJ Zhang X Kumar A Atkinson EJ Zhu Y Jachim S. The Senescence-Associated Secretome as an Indicator of Age and Medical Risk. JCI Insight (2020) 5(12):e133668. doi: 10.1172/jci.insight.133668 PMC7406245 32554926

[51] El-Galaly TC Villa D Alzahrani M Hansen JW Sehn LH Wilson D. Outcome Prediction by Extranodal Involvement, IPI, R-IPI, and NCCN-IPI in the PET/CT and Rituximab Era: A Danish-Canadian Study of 443 Patients With Diffuse-Large B-Cell Lymphoma. Am J Hematol (2015) 90(11):1041–6. doi: 10.1002/ajh.24169 26260224

[52] Ennishi D Mottok A Ben-Neriah S Shulha HP Farinha P Chan FC. Genetic Profiling of MYC and BCL2 in Diffuse Large B-Cell Lymphoma Determines Cell-of-Origin-Specific Clinical Impact. Blood (2017) 129(20):2760–70. doi: 10.1182/blood-2016-11-747022 28351934

[53] Keane C Gill D Vari F Cross D Griffiths L Gandhi M. CD4(+) Tumor Infiltrating Lymphocytes are Prognostic and Independent of R-IPI in Patients With DLBCL Receiving R-CHOP Chemo-Immunotherapy. Am J Hematol (2013) 88(4):273–6. doi: 10.1002/ajh.23398 23460351

[54] Montalban C Diaz-Lopez A Dlouhy I Rovira J Lopez-Guillermo A Alonso S. Validation of the NCCN-IPI for Diffuse Large B-Cell Lymphoma (DLBCL): The Addition of Beta2 -Microglobulin Yields a More Accurate GELTAMO-IPI. Br J Haematol (2017) 176(6):918–28. doi: 10.1111/bjh.14489 28106247

[55] Scott DW Mottok A Ennishi D Wright GW Farinha P Ben-Neriah S. Prognostic Significance of Diffuse Large B-Cell Lymphoma Cell of Origin Determined by Digital Gene Expression in Formalin-Fixed Paraffin-Embedded Tissue Biopsies. J Clin Oncol (2015) 33(26):2848–56. doi: 10.1200/JCO.2014.60.2383 PMC4554747 26240231

[56] Han Y Yang J Liu P He X Zhang C Zhou S. Prognostic Nomogram for Overall Survival in Patients With Diffuse Large B-Cell Lymphoma. Oncologist (2019) 24(11):e1251–e61. doi: 10.1634/theoncologist.2018-0361 PMC6853087 30952824

[57] Li M Liu CL Yin WJ He YX Xue XM Duan ZJ. The Clinical Significance of a New Classification Algorithm in Chinese DLBCL Cases. Zhonghua Xue Ye Xue Za Zhi (2012) 33(10):801–4. doi: 10.3760/cma.j.issn.0253-2727.2012.10.003 23384897

[58] Liu Y Wang X Ding N Mi L Ping L Jin X. TP53 Arg72 as a Favorable Prognostic Factor for Chinese Diffuse Large B-Cell Lymphoma Patients Treated With CHOP. BMC Cancer (2017) 17(1):743. doi: 10.1186/s12885-017-3760-0 29126407 PMC5680759

[59] Ren W Ye X Su H Li W Liu D Pirmoradian M. Genetic Landscape of Hepatitis B Virus-Associated Diffuse Large B-Cell Lymphoma. Blood (2018) 131(24):2670–81. doi: 10.1182/blood-2017-11-817601 PMC6063049 29545328

[60] Shi Y Han Y Yang J Liu P He X Zhang C. Clinical Features and Outcomes of Diffuse Large B-Cell Lymphoma Based on Nodal or Extranodal Primary Sites of Origin: Analysis of 1,085 WHO Classified Cases in a Single Institution in China. Chin J Cancer Res (2019) 31(1):152–61. doi: 10.21147/j.issn.1000-9604.2019.01.10 PMC6433587 30996573

