# Supplementary figures and images for: Oncogenic Mutations and Tumor Microenvironment Alterations of Older Patients With Diffuse Large B-Cell Lymphoma
Source: Front Immunol. 2022 Mar 25;13:842439. doi: 10.3389/fimmu.2022.842439 (PMC8990904; doi:10.3389/fimmu.2022.842439)

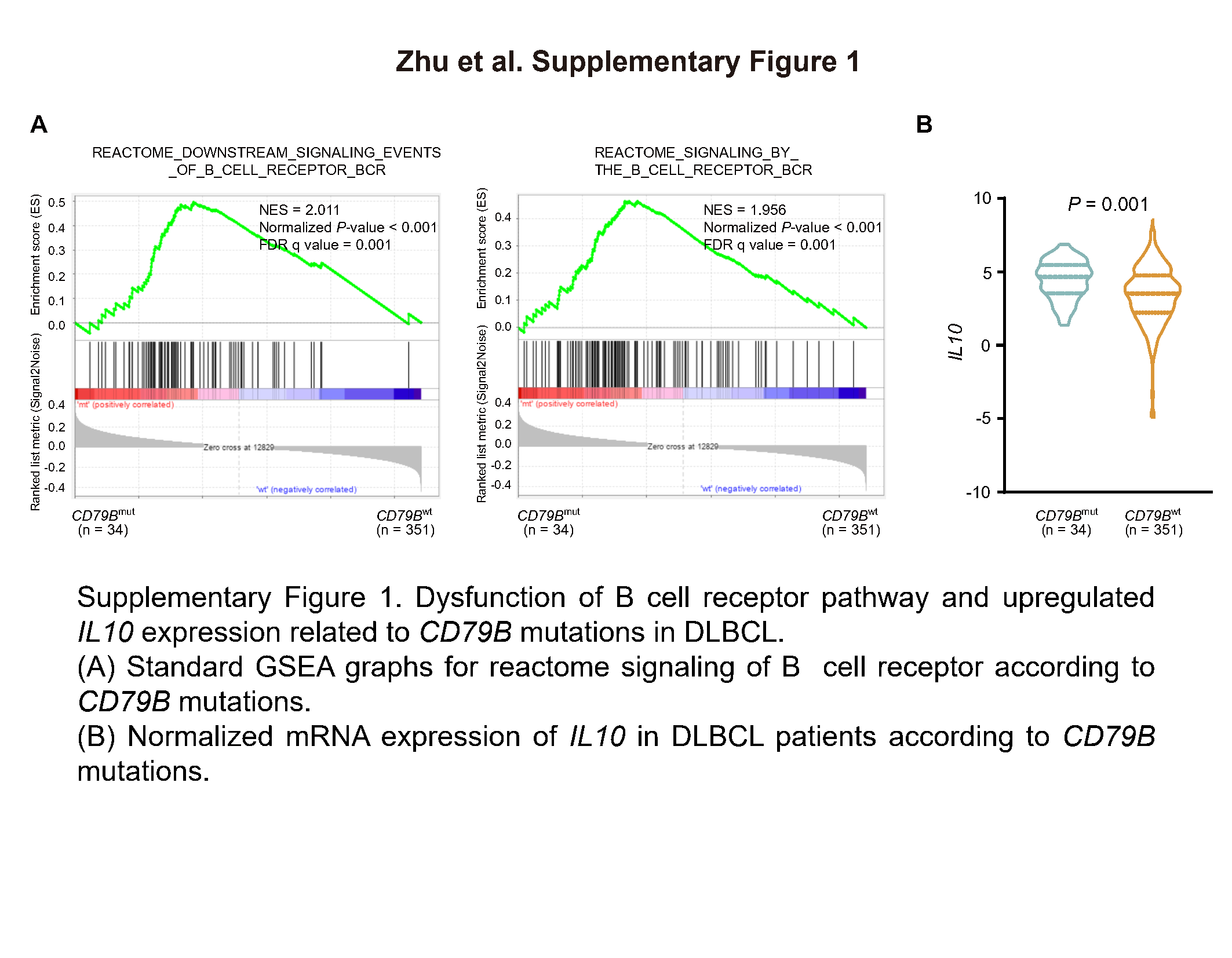

Supplement: Supplementary file 1 [file Image_1.tiff]

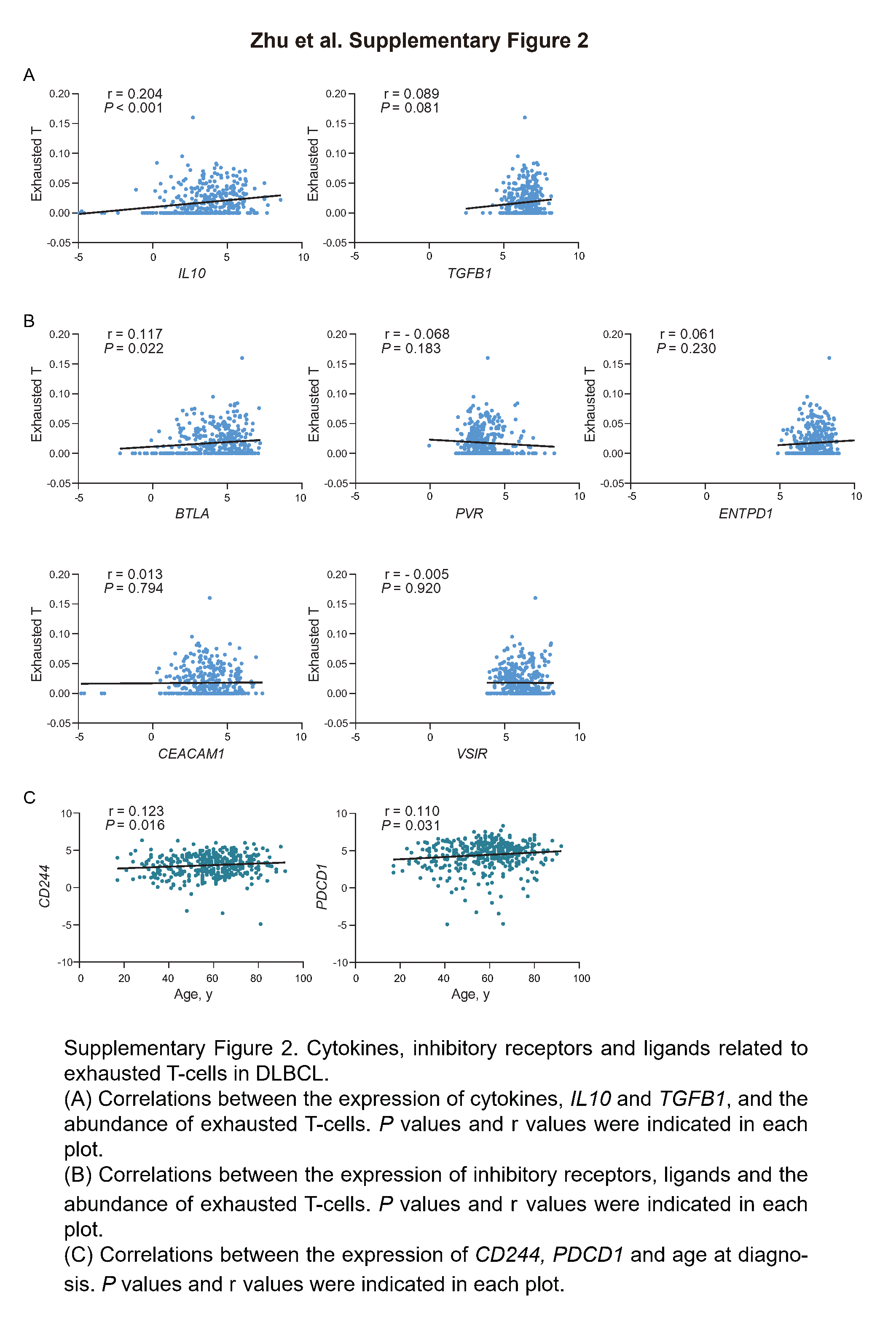

Supplement: Supplementary file 2 [file Image_2.tiff]

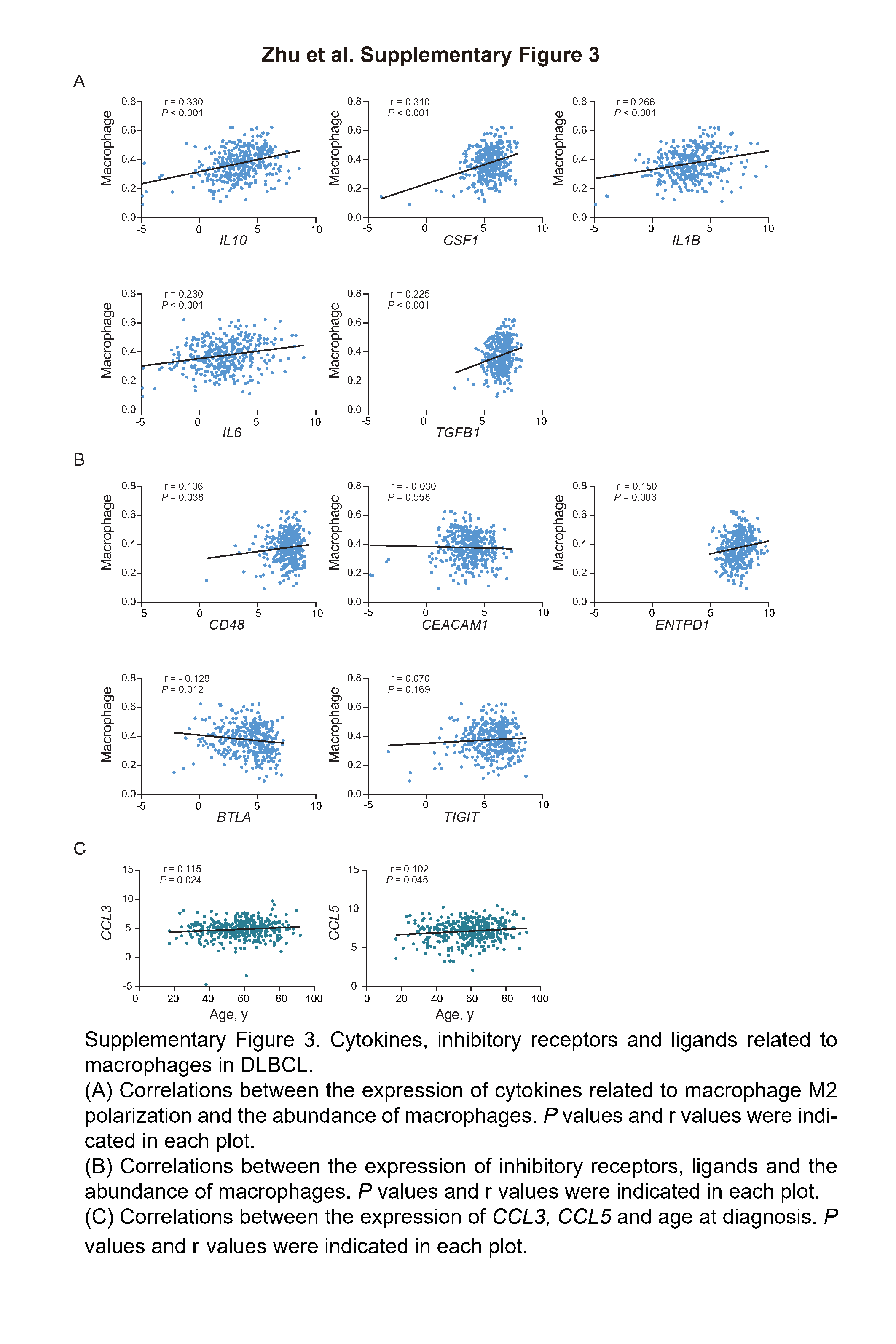

Supplement: Supplementary file 3 [file Image_3.tiff]
