# Supplementary material for: Oncogenic Mutations and Tumor Microenvironment Alterations of Older Patients With Diffuse Large B-Cell Lymphoma
Source: Front Immunol. 2022 Mar 25;13:842439. doi: 10.3389/fimmu.2022.842439 (PMC8990904; doi:10.3389/fimmu.2022.842439)
Supplement: Supplementary file 5 [file Table_2.docx]

Supplementary Table 2

Clinical and pathological characteristics among patients with DNA sequencing data according to DNA sequencing methods (n = 1150)

| Characteristics |  | WGS  (n = 117) | WES  (n = 223) | Targeted sequencing  (n = 810) | *P* value | *P* value ^a^ | P value ^b^ | *P* value ^c^ |
| --- | --- | --- | --- | --- | --- | --- | --- | --- |
| Gender |  |  |  |  |  |  |  |  |
|  | Male | 68 (58.12%) | 120 (53.81%) | 439 (54.20%) | 0.708 |  |  |  |
|  | Female | 49 (41.88%) | 103 (46.19%) | 371 (45.80%) |  |  |  |  |
| Age |  |  |  |  |  |  |  |  |
|  | ≤ 60 y | 52 (44.44%) | 155 (69.51%) | 409 (50.49%) | < 0.001 | < 0.001 | 0.221 | <0.001 |
|  | > 60 y | 65 (55.56%) | 68 (30.49%) | 401 (49.51%) |  |  |  |  |
| Ann Arbor stage | |  |  |  |  |  |  |  |
|  | I-II | 48 (41.03%) | 119 (53.36%) | 409 (50.49%) | 0.088 |  |  |  |
|  | III-IV | 69 (58.97%) | 104 (46.64%) | 401 (49.51%) |  |  |  |  |
| LDH |  |  |  |  |  |  |  |  |
|  | Normal | 48 (41.03%) | 128 (57.40%) | 389 (48.02%) | 0.008 | 0.004 | 0.156 | 0.013 |
|  | Elevated | 69 (58.97%) | 95 (42.60%) | 421 (51.98%) |  |  |  |  |
| ECOG score | |  |  |  |  |  |  |  |
|  | 0-1 | 101 (86.32%) | 206 (92.38%) | 708 (87.41%) | 0.098 |  |  |  |
|  | ≥2 | 16 (13.68%) | 17 (7.62%) | 102 (12.59%) |  |  |  |  |
| Extranodal involvement | |  |  |  |  |  |  |  |
|  | 0-1 | 71 (60.68%) | 164 (73.54%) | 566 (69.88%) | 0.048 | 0.015 | 0.045 | 0.287 |
|  | ≥2 | 46 (39.32%) | 59 (26.46%) | 244 (30.12%) |  |  |  |  |
| Cell of origin (Hans) | |  |  |  |  |  |  |  |
|  | GCB | 38/115 (33.04%) | 79/222 (35.59%) | 324/758 (42.74%) | 0.040 | 0.642 | 0.049 | 0.057 |
|  | Non-GCB | 77/115 (66.96%) | 143/222 (64.41%) | 434/758 (57.26%) |  |  |  |  |
| Double expressor | |  |  |  |  |  |  |  |
|  | Yes | 33/115 (28.70%) | 54/222 (24.32%) | 216/762 (28.35%) | 0.479 |  |  |  |
|  | No | 82/115 (71.30%) | 168/222 (75.68%) | 546/762 (71.65%) |  |  |  |  |
| Double-hit/triple-hit | |  |  |  |  |  |  |  |
|  | Yes | 6/52 (11.54%) | 3/85 (3.53%) | 34/689 (4.93%) | 0.110 |  |  |  |
|  | No | 46/52 (88.46%) | 82/85 (96.47%) | 655/689 (95.07%) |  |  |  |  |

*P* value indicated difference between the patients with different DNA sequencing methods.

a *P* value indicated difference between patients with WGS data and patients with WES data.

b *P* value indicated difference between patients with WGS data and patients with targeted sequencing patients.

c *P* value indicated difference between patients with WES data and patients with targeted sequencing patients.

Abbreviations: WGS, whole genome sequencing; WES, whole exome sequencing; LDH, lactate dehydrogenase; ECOG, Eastern Cooperative Oncology Group; GCB, germinal center B-cell.
