# Supplementary material for: Oncogenic Mutations and Tumor Microenvironment Alterations of Older Patients With Diffuse Large B-Cell Lymphoma
Source: Front Immunol. 2022 Mar 25;13:842439. doi: 10.3389/fimmu.2022.842439 (PMC8990904; doi:10.3389/fimmu.2022.842439)
Supplement: Supplementary file 6 [file Table_3.docx]

Supplementary Table 3

Clinical and pathological characteristics among patients with DNA sequencing data according to DNA sequencing methods after balancing the baseline using propensity score matching (n = 404)

| Characteristics | | WGS (n = 65) | WES (n = 107) | Targeted sequencing  (n = 232) | *P* value |
| --- | --- | --- | --- | --- | --- |
| Gender |  |  |  |  |  |
|  | Male | 39 (60.00%) | 61 (57.01%) | 118 (50.86%) | 0.324 |
|  | Female | 26 (40.00%) | 46 (42.99%) | 114 (49.14%) |  |
| Age |  |  |  |  |  |
|  | ≤ 60 y | 33 (50.77%) | 62 (57.94%) | 104 (44.83%) | 0.078 |
|  | > 60 y | 32 (49.23%) | 45 (42.06%) | 128 (55.17%) |  |
| Ann Arbor stage | |  |  |  |  |
|  | I-II | 35 (53.85%) | 58 (54.21%) | 112 (48.28%) | 0.515 |
|  | III-IV | 30 (46.15%) | 49 (45.79%) | 120 (51.72%) |  |
| LDH |  |  |  |  |  |
|  | Normal | 33 (50.77%) | 62 (57.94%) | 105 (45.26%) | 0.092 |
|  | Elevated | 32 (49.23%) | 45 (42.06%) | 127 (54.74%) |  |
| ECOG score | |  |  |  |  |
|  | 0-1 | 57 (87.69%) | 98 (91.59%) | 202 (87.07%) | 0.475 |
|  | ≥2 | 8 (12.31%) | 9 (8.41%) | 30 (12.93%) |  |
| Extranodal involvement | | |  |  |  |
|  | 0-1 | 48 (73.85%) | 79 (73.83%) | 166 (71.55%) | 0.879 |
|  | ≥2 | 17 (26.15%) | 28 (26.17%) | 66 (28.45%) |  |
| Cell of origin (Hans) | | |  |  |  |
|  | GCB | 23/64 (35.94%) | 37 (34.58%) | 94/226 (41.59%) | 0.414 |
|  | Non-GCB | 41/64 (64.06%) | 70 (65.42%) | 132/226 (58.41%) |  |
| Double expressor | |  |  |  |  |
|  | Yes | 21/64 (32.81%) | 35 (32.71%) | 58/224 (25.89%) | 0.330 |
|  | No | 43/64 (67.19%) | 72 (67.29%) | 166/224 (74.11%) |  |
| Double-hit/triple-hit | | |  |  |  |
|  | Yes | 4/28 (14.29%) | 2/31 (6.45%) | 7/185 (3.78%) | 0.052 |
|  | No | 24/28 (85.71%) | 29/31 (93.55%) | 178/185 (96.22%) |  |

*P* value indicated difference between the patients with different DNA sequencing methods.

Abbreviations: WGS, whole genome sequencing; WES, whole exome sequencing; LDH, lactate dehydrogenase; ECOG, Eastern Cooperative Oncology Group; GCB, germinal center B-cell.
