# Supplementary material for: Oncogenic Mutations and Tumor Microenvironment Alterations of Older Patients With Diffuse Large B-Cell Lymphoma
Source: Front Immunol. 2022 Mar 25;13:842439. doi: 10.3389/fimmu.2022.842439 (PMC8990904; doi:10.3389/fimmu.2022.842439)
Supplement: Supplementary file 7 [file Table_4.docx]

Supplementary Table 4

Frequencies of mutations according to DNA sequencing methods among patients in Supplementary Table 3 (n = 404)

| Gene | WGS  (n = 65) | WES  (n = 107) | Targeted sequencing  (n = 232) | *P* value-adjusted |  |
| --- | --- | --- | --- | --- | --- |
| *ARID1A* | 5 (7.69%) | 4 (3.74%) | 14 (6.03%) | 1.000 |  |
| *ATM* | 6 (9.23%) | 9 (8.41%) | 21 (9.05%) | 1.000 |  |
| *B2M* | 11 (16.92%) | 6 (5.61%) | 11 (4.74%) | 0.356 |  |
| *BCL6* | 5 (7.69%) | 5 (4.67%) | 10 (4.31%) | 1.000 |  |
| *BTG1* | 12 (18.46%) | 13 (12.15%) | 22 (9.48%) | 1.000 |  |
| *BTG2* | 11 (16.92%) | 17 (15.89%) | 40 (17.24%) | 1.000 |  |
| *CARD11* | 4 (6.15%) | 7 (6.54%) | 22 (9.48%) | 1.000 |  |
| *CCND3* | 8 (12.31%) | 6 (5.61%) | 21 (9.05%) | 1.000 |  |
| *CD58* | 3 (4.62%) | 6 (5.61%) | 11 (4.74%) | 1.000 |  |
| *CD70* | 7 (10.77%) | 11 (10.28%) | 18 (7.76%) | 1.000 |  |
| *CD79A* | 2 (3.08%) | 0 (0.00%) | 9 (3.88%) | 1.000 |  |
| *CD79B* | 6 (9.23%) | 9 (8.41%) | 37 (15.95%) | 1.000 |  |
| *CIITA* | 4 (6.15%) | 5 (4.67%) | 6 (2.59%) | 1.000 |  |
| *CREBBP* | 0 (0.00%) | 10 (9.35%) | 21 (9.05%) | 0.770 |  |
| *DDX3X* | 6 (9.23%) | 13 (12.15%) | 5 (2.16%) | < 0.001 |  |
| *DTX1* | 13 (20.00%) | 14 (13.08%) | 34 (14.66%) | 1.000 |  |
| *DUSP2* | 7 (10.77%) | 2 (1.87%) | 14 (6.03%) | 1.000 |  |
| *EBF1* | 5 (7.69%) | 10 (9.35%) | 9 (3.88%) | 1.000 |  |
| *EP300* | 5 (7.69%) | 11 (10.28%) | 10 (4.31%) | 1.000 |  |
| *EZH2* | 1 (1.5%) | 4 (3.70%) | 21 (9.10%) | 1.000 |  |
| *FAS* | 4 (6.15%) | 10 (9.35%) | 11 (4.74%) | 1.000 |  |
| *FBXW7* | 1 (1.54%) | 3 (2.80%) | 2 (0.86%) | 1.000 |  |
| *FOXO1* | 3 (4.62%) | 7 (6.54%) | 8 (3.45%) | 1.000 |  |
| *GNA13* | 6 (9.23%) | 1 (0.93%) | 11 (4.74%) | 1.000 |  |
| *HIST1H1C* | 5 (7.69%) | 8 (7.48%) | 10 (4.31%) | 1.000 |  |
| *HIST1H1E* | 11 (16.92%) | 11 (10.28%) | 28 (12.07%) | 1.000 |  |
| *IRF4* | 1 (1.54%) | 7 (6.54%) | 11 (4.74%) | 1.000 |  |
| *IRF8* | 2 (3.08%) | 3 (2.80%) | 5 (2.16%) | 1.000 |  |
| *KMT2C* | 3 (4.62%) | 11 (10.28%) | 23 (9.91%) | 1.000 |  |
| *KMT2D* | 15 (23.08%) | 26 (24.30%) | 51 (21.98%) | 1.000 |  |
| *LYN* | 3 (4.60%) | 2 (1.90%) | 6 (2.60%) | 1.000 |  |
| *MAPK7* | 0 (0.00%) | 3 (2.80%) | 4 (1.72%) | 1.000 |  |
| *MPEG1* | 5 (7.69%) | 8 (7.48%) | 21 (9.05%) | 1.000 |  |
| *MTOR* | 1 (1.54%) | 3 (2.80%) | 2 (0.86%) | 1.000 |  |
| *MYC* | 4 (6.15%) | 4 (3.74%) | 16 (6.90%) | 1.000 |  |
| *MYD88* | 11 (16.92%) | 15 (14.02%) | 51 (21.98%) | 1.000 |  |
| *NFKBIE* | 3 (4.62%) | 5 (4.67%) | 14 (6.03%) | 1.000 |  |
| *NOTCH1* | 2 (3.08%) | 4 (3.74%) | 14 (6.03%) | 1.000 |  |
| *NOTCH2* | 4 (6.15%) | 8 (7.48%) | 16 (6.90%) | 1.000 |  |
| *PIM1* | 11 (16.92%) | 23 (21.50%) | 60 (25.86%) | 1.000 |  |
| *PRDM1* | 5 (7.70%) | 9 (8.40%) | 22 (9.50%) | 1.000 |  |
| *PTPN6* | 4 (6.15%) | 5 (4.67%) | 13 (5.60%) | 1.000 |  |
| *SGK1* | 5 (7.69%) | 6 (5.61%) | 14 (6.03%) | 1.000 |  |
| *SOCS1* | 7 (10.77%) | 9 (8.41%) | 25 (10.78%) | 1.000 |  |
| *STAT3* | 7 (10.77%) | 5 (4.67%) | 10 (4.31%) | 1.000 |  |
| *STAT6* | 1 (1.54%) | 0 (0.00%) | 5 (2.16%) | 1.000 |  |
| *TBL1XR1* | 5 (7.69%) | 7 (6.54%) | 16 (6.90%) | 1.000 |  |
| *TET2* | 8 (12.31%) | 15 (14.02%) | 30 (12.93%) | 1.000 |  |
| *TMSB4X* | 6 (9.20%) | 13 (12.10%) | 14 (6.00%) | 1.000 |  |
| *TNFAIP3* | 5 (7.69%) | 5 (4.67%) | 25 (10.78%) | 1.000 |  |
| *TNFRSF14* | 8 (12.31%) | 5 (4.67%) | 5 (2.16%) | 0.220 |  |
| *TP53* | 9 (13.85%) | 14 (13.08%) | 29 (12.50%) | 1.000 |  |
| *TSC2* | 0 (0.00%) | 7 (6.54%) | 6 (2.59%) | 1.000 |  |
| *ZFP36L1* | 3 (4.62%) | 7 (6.54%) | 11 (4.74%) | 1.000 |  |
| *ZNF608* | 5 (7.69%) | 5 (4.67%) | 17 (7.33%) | 1.000 |  |

*P* value indicated difference between the DNA sequencing methods.

Abbreviations: WGS, whole genome sequencing; WES, whole exome sequencing.
