# Supplementary material for: Oncogenic Mutations and Tumor Microenvironment Alterations of Older Patients With Diffuse Large B-Cell Lymphoma
Source: Front Immunol. 2022 Mar 25;13:842439. doi: 10.3389/fimmu.2022.842439 (PMC8990904; doi:10.3389/fimmu.2022.842439)
Supplement: Supplementary file 8 [file Table_5.docx]

Supplementary Table 5

Gene lists according to Biology Processes

| Biology Processes |  | Genes |
| --- | --- | --- |
| Chromatin organization | |  |
|  | Histone/DNA methylation | *EZH2, HIST1H1C, HIST1H1E, KMT2C, KMT2D, TET2* |
|  | Histone acetylation | *CREBBP, EP300, TBL1XR1* |
|  | Chromatin remodeling | *ARID1A, SGK1* |
| Immune response | |  |
|  | T cell activation | *BCL6, CD70, MPEG1, PRDM1, PTPN6, TMSB4X, TNFRSF14, ZFP36L1* |
|  | B cell differentiation | *BCL6, CARD11, CD79A, CD79B, NOTCH2, PTPN6, ZFP36L1* |
|  | Response to interferon-gamma | *B2M, CD58, CIITA, IRF4, IRF8, SOCS1* |
| Cell cycle/p53 | |  |
|  | Cell cycle/p53 | *ATM, BTG1, BTG2, CCND3, DTX1, EBF1, FAS, FBXW7, MAPK7, MYC, NFKBIE, TP53* |
| Signaling pathway | |  |
|  | Wnt | *DDX3X, FOXO1, GNA13, IRF4, PIM1, TBL1XR1, TSC2* |
|  | BCR/NF-κB | *CARD11, CD79A, CD79B, DTX1, LYN, MYD88, PIM1, PTPN6, TNFAIP3, ZNF608* |
|  | JAK-STAT | *BCL6, DUSP2, SOCS1, STAT3, STAT6* |
|  | PI3K-AKT | *ATM, DDX3X, GNA13, IRF4, MTOR, NOTCH1, TSC2* |

Abbreviations: BCR, B-cell receptor.
