# Supplementary material for: Oncogenic Mutations and Tumor Microenvironment Alterations of Older Patients With Diffuse Large B-Cell Lymphoma
Source: Front Immunol. 2022 Mar 25;13:842439. doi: 10.3389/fimmu.2022.842439 (PMC8990904; doi:10.3389/fimmu.2022.842439)
Supplement: Supplementary file 9 [file Table_6.docx]

Supplementary Table 6

Clinical and pathological characteristics among patients with WGS data according to with or without RNA sequencing data (n = 117)

| Characteristics | | With RNA sequencing data (n = 98) | Without RNA sequencing data  (n = 19) | *P* value |
| --- | --- | --- | --- | --- |
| Gender |  |  |  |  |
|  | Male | 54 (55.10%) | 14 (73.68%) | 0.133 |
|  | Female | 44 (44.90%) | 5 (26.32%) |  |
| Age |  |  |  |  |
|  | ≤ 60 y | 44 (44.90%) | 8 (42.11%) | 0.823 |
|  | > 60 y | 54 (55.10%) | 11 (57.89%) |  |
| Ann Arbor stage | |  |  |  |
|  | I-II | 41 (41.84%) | 7 (36.84%) | 0.685 |
|  | III-IV | 57 (58.16%) | 12 (63.16%) |  |
| LDH |  |  |  |  |
|  | Normal | 41 (41.84%) | 7 (36.84%) | 0.685 |
|  | Elevated | 57 (58.16%) | 12 (63.16%) |  |
| ECOG score | |  |  |  |
|  | 0-1 | 85 (86.73%) | 16 (84.21%) | 0.723 |
|  | ≥2 | 13 (13.27%) | 3 (15.79%) |  |
| Extranodal involvement | | |  |  |
|  | 0-1 | 59 (60.20%) | 12 (63.16%) | 0.809 |
|  | ≥2 | 39 (39.80%) | 7 (36.84%) |  |
| Cell of origin (Hans) | | |  |  |
|  | GCB | 29/96 (30.21%) | 9 (47.37%) | 0.146 |
|  | Non-GCB | 67/96 (69.79%) | 10 (52.63%) |  |
| Double expressor | |  |  |  |
|  | Yes | 30 (30.61%) | 3/17 (17.65%) | 0.388 |
|  | No | 68 (69.39%) | 14/17 (82.35%) |  |
| Double-hit/triple-hit | | |  |  |
|  | Yes | 5/49 (10.20%) | 1/3 (33.33%) | 0.313 |
|  | No | 44/49 (89.80%) | 2/3 (66.67%) |  |

*P* value indicated difference between the patients with or without RNA sequencing data.

Abbreviations: WGS, whole genome sequencing; LDH, lactate dehydrogenase; ECOG, Eastern Cooperative Oncology Group; GCB, germinal center B-cell.
