# Supplementary material for: Oncogenic Mutations and Tumor Microenvironment Alterations of Older Patients With Diffuse Large B-Cell Lymphoma
Source: Front Immunol. 2022 Mar 25;13:842439. doi: 10.3389/fimmu.2022.842439 (PMC8990904; doi:10.3389/fimmu.2022.842439)
Supplement: Supplementary file 10 [file Table_7.docx]

Supplementary Table 7

Clinical and pathological characteristics among patients with WES data according to with or without RNA sequencing data (n = 223)

| Characteristics | | With RNA sequencing data (n = 98) | Without RNA sequencing data  (n = 125) | *P* value |
| --- | --- | --- | --- | --- |
| Gender |  |  |  |  |
|  | Male | 54 (55.10%) | 66 (52.80%) | 0.732 |
|  | Female | 44 (44.90%) | 59 (47.20%) |  |
| Age |  |  |  |  |
|  | ≤ 60 y | 67 (68.37%) | 88 (70.40%) | 0.743 |
|  | > 60 y | 31 (31.63%) | 37 (29.60%) |  |
| Ann Arbor stage | |  |  |  |
|  | I-II | 50 (51.02%) | 69 (55.20%) | 0.535 |
|  | III-IV | 48 (48.98%) | 56 (44.80%) |  |
| LDH |  |  |  |  |
|  | Normal | 52 (53.06%) | 76 (60.80%) | 0.246 |
|  | Elevated | 46 (46.94%) | 49 (39.20%) |  |
| ECOG score | |  |  |  |
|  | 0-1 | 90 (91.84%) | 116 (92.80%) | 0.788 |
|  | ≥2 | 8 (8.16%) | 9 (7.20%) |  |
| Extranodal involvement | | |  |  |
|  | 0-1 | 72 (73.47%) | 92 (73.60%) | 0.982 |
|  | ≥2 | 26 (26.53%) | 33 (26.40%) |  |
| Cell of origin (Hans) | | |  |  |
|  | GCB | 28/97 (28.87%) | 51/125 (40.80%) | 0.065 |
|  | Non-GCB | 69/97 (71.13%) | 74/125 (59.20%) |  |
| Double expressor | |  |  |  |
|  | Yes | 20/97 (20.62%) | 34/125 (27.20%) | 0.257 |
|  | No | 77/97 (79.38%) | 91/125 (72.80%) |  |
| Double-hit/triple-hit | | |  |  |
|  | Yes | 1/65 (1.54%) | 2/20 (10.00%) | 0.137 |
|  | No | 64/65 (98.46%) | 18/20 (90.00%) |  |

*P* value indicated difference between the patients with or without RNA sequencing data.

Abbreviations: WES, whole exome sequencing; LDH, lactate dehydrogenase; ECOG, Eastern Cooperative Oncology Group; GCB, germinal center B-cell.
