# Supplementary material for: Oncogenic Mutations and Tumor Microenvironment Alterations of Older Patients With Diffuse Large B-Cell Lymphoma
Source: Front Immunol. 2022 Mar 25;13:842439. doi: 10.3389/fimmu.2022.842439 (PMC8990904; doi:10.3389/fimmu.2022.842439)
Supplement: Supplementary file 11 [file Table_8.docx]

Supplementary Table 8

Clinical and pathological characteristics among patients with targeted sequencing data according to with or without RNA sequencing data (n = 810)

| Characteristics | | With RNA sequencing data (n = 189) | Without RNA sequencing data  (n = 621) | *P* value |
| --- | --- | --- | --- | --- |
| Gender |  |  |  |  |
|  | Male | 116 (61.38%) | 323 (52.01%) | 0.024 |
|  | Female | 73 (38.62%) | 298 (47.99%) |  |
| Age |  |  |  |  |
|  | ≤ 60 y | 91 (48.15%) | 318 (51.21%) | 0.461 |
|  | > 60 y | 98 (51.85%) | 303 (48.79%) |  |
| Ann Arbor stage | |  |  |  |
|  | I-II | 86 (45.50%) | 323 (52.01%) | 0.117 |
|  | III-IV | 103 (54.50%) | 298 (47.99%) |  |
| LDH |  |  |  |  |
|  | Normal | 57 (30.16%) | 332 (53.46%) | < 0.001 |
|  | Elevated | 132 (69.84%) | 289 (46.54%) |  |
| ECOG score | |  |  |  |
|  | 0-1 | 166 (87.83%) | 542 (87.28%) | 0.841 |
|  | ≥2 | 23 (12.17%) | 79 (12.72%) |  |
| Extranodal involvement | | |  |  |
|  | 0-1 | 125 (66.14%) | 441 (71.01%) | 0.201 |
|  | ≥2 | 64 (33.86%) | 180 (28.99%) |  |
| Cell of origin (Hans) | | |  |  |
|  | GCB | 78/182 (42.86%) | 246/576 (42.71%) | 0.972 |
|  | Non-GCB | 104/182 (57.14%) | 330/576 (57.29%) |  |
| Double expressor | |  |  |  |
|  | Yes | 35/186 (18.82%) | 181/576 (31.42%) | 0.001 |
|  | No | 151/186 (81.18%) | 395/576 (68.58%) |  |
| Double-hit/triple-hit | | |  |  |
|  | Yes | 6/171 (3.51%) | 28/518 (5.41%) | 0.321 |
|  | No | 165/171 (96.49%) | 490/518 (94.59%) |  |

*P* value indicated difference between the patients with or without RNA sequencing data.

Abbreviations: LDH, lactate dehydrogenase; ECOG, Eastern Cooperative Oncology Group; GCB, germinal center B-cell.
