# Supplementary material for: Oncogenic Mutations and Tumor Microenvironment Alterations of Older Patients With Diffuse Large B-Cell Lymphoma
Source: Front Immunol. 2022 Mar 25;13:842439. doi: 10.3389/fimmu.2022.842439 (PMC8990904; doi:10.3389/fimmu.2022.842439)
Supplement: Supplementary file 13 [file Table_10.docx]

**Supplementary Table 10**

Fluorescence in-situ hybridization of DLBCL patients (n = 1064)

|  | Age | | *P* value |
| --- | --- | --- | --- |
|  | ≤ 60 y (n = 529) | > 60 y (n = 535) |  |
| Translocation |  |  | 0.505 |
| Triple-hit | 2 (0.38%) | 3 (0.56%) |  |
| MYC/BCL2 double-hit | 15 (2.84%) | 13 (2.43%) |  |
| MYC/BCL6 double-hit | 14 (2.65%) | 9 (1.68%) |  |
| MYC single hit | 13 (2.46%) | 21 (3.93%) |  |
| MYC translocation-negative | 485 (91.68%) | 489 (91.40%) |  |
